# Late‐onset acute type 1 diabetes mellitus 7 months after discontinuation of pembrolizumab against lung cancer

**DOI:** 10.1111/1759-7714.14736

**Published:** 2022-11-21

**Authors:** Seiya Ichihara, Michihiro Kunishige, Naoki Kadota, Yoshio Okano, Hisanori Machida, Nobuo Hatakeyama, Keishi Naruse, Tsutomu Shinohara, Eiji Takeuchi

**Affiliations:** ^1^ Department of Respiratory Medicine National Hospital Organization Kochi Hospital Kochi Japan; ^2^ Department of Pathology National Hospital Organization Kochi Hospital Kochi Japan; ^3^ Department of Community Medicine for Respirology Graduate School of Biomedical Sciences, Tokushima University Tokushima Japan; ^4^ Department of Clinical Investigation National Hospital Organization Kochi Hospital Kochi Japan

**Keywords:** acute‐onset type 1 diabetes, after discontinuation, immune‐related adverse events, late‐onset, non‐small‐cell lung cancer

## Abstract

Immune‐related adverse events (irAEs) occur in rare cases, even after the completion of immune checkpoint inhibitor (ICI) therapy. We encountered a lung cancer patient diagnosed with acute‐onset type 1 diabetes mellitus (DM) 7 months after the cessation of ICI. A 68‐year‐old woman was referred to our hospital for chest abnormalities. She was diagnosed with lung adenocarcinoma cT4N2M1c, stage IVB. Immunostaining showed that the expression of programmed death ligand 1 in tumor cells was negative. A genetic analysis using the Oncomine Dx Target Test Multi‐CDx System revealed that the primary tumor was positive for ERBB2. Combined immunotherapy with carboplatin, pemetrexed, and pembrolizumab was performed as first‐line therapy, followed by maintenance therapy with pemetrexed plus pembrolizumab, which was successful. After the seventh course, maintenance therapy was stopped because only the primary tumor showed local enlargement. Local chest radiotherapy (66 Gy/33 Fr) was performed, and the patient was followed up. HbA1c was 4.9% 3 months after the completion of pembrolizumab, and dry mouth and polyuria occurred after 5 months. Seven months later, the patient developed diabetic ketoacidosis with a blood glucose of 348 mg/dL and an HbA1c of 11.3%. Antiglutamic acid decarboxylase antibodies were negative and urinary C‐peptide was 9.3 μg/day. The patient was diagnosed with acute‐onset type 1 diabetes and received insulin therapy. There has been no case report of type 1 diabetes diagnosed 7 months after the last administration of an ICI. These results indicate that irAE needs to be considered even after the cessation of ICI.

## Graphical abstract

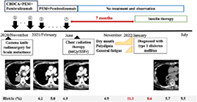

## INTRODUCTION

Immune checkpoint inhibitors (ICIs) have markedly changed the treatment of lung cancer; however, severe immune‐related adverse events (irAEs) occur in rare cases,^1^ even after the discontinuation of ICIs.^2^, ^3^, ^4^, ^5^, ^6^, ^7^, ^8^ We encountered a lung cancer patient diagnosed with acute‐onset type 1 diabetes mellitus (DM) 7 months after the cessation of pembrolizumab. We herein present this case.

## CASE REPORT

**FIGURE 1:**
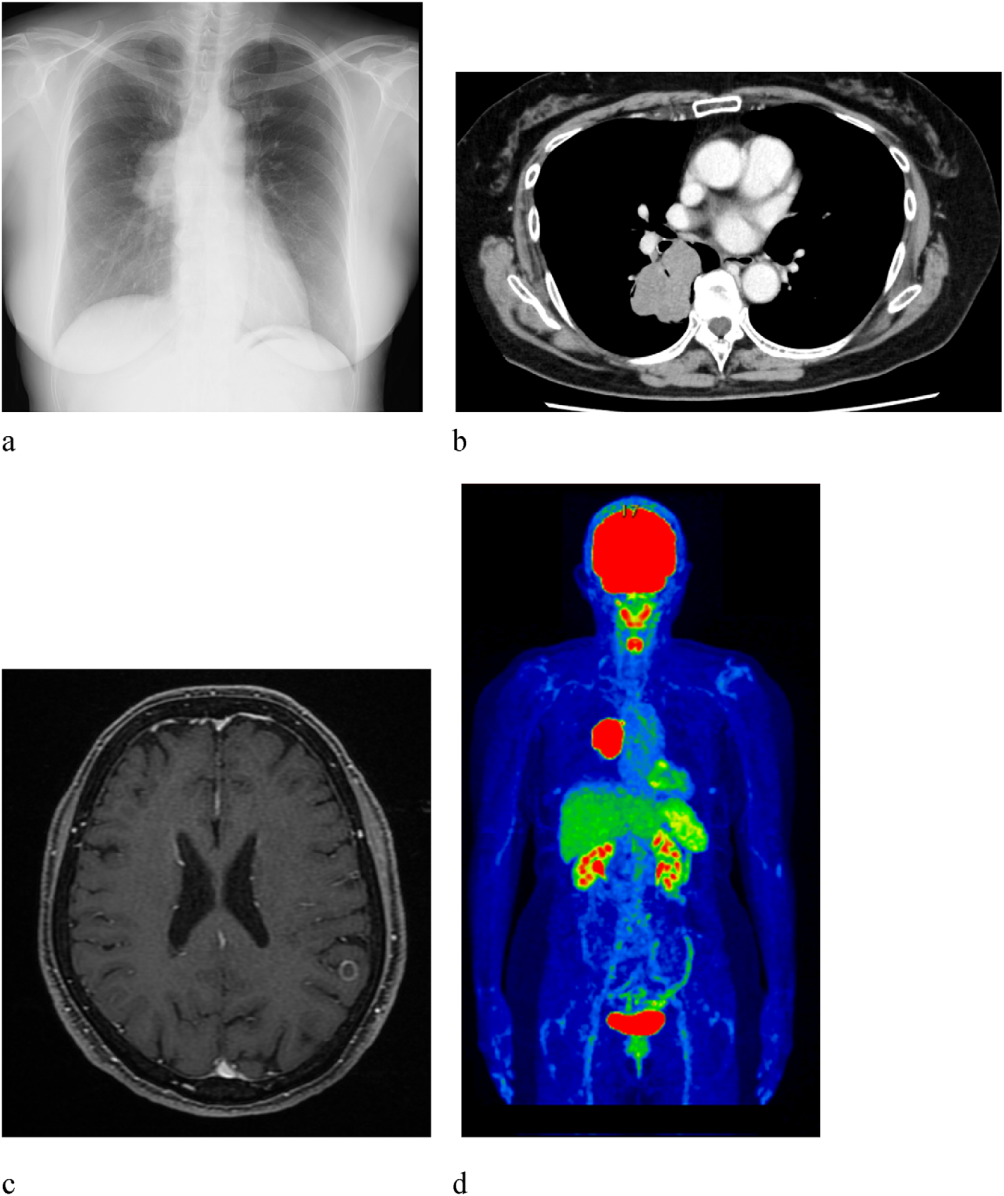
(a) Chest X‐ray, (b) contrast‐enhanced computed tomography (CT), (c) contrast‐enhanced head magnetic resonance imaging (MRI), and (d) positron emission tomography/computed tomography (PET/CT) at first admission. An irregular tumor was present in the right lower lobe, a metastatic brain tumor was detected in the left parietal lobe, and fluorodeoxyglucose uptake was noted. The maximum standardized uptake value (SUVmax) was 19.2

**FIGURE 2:**
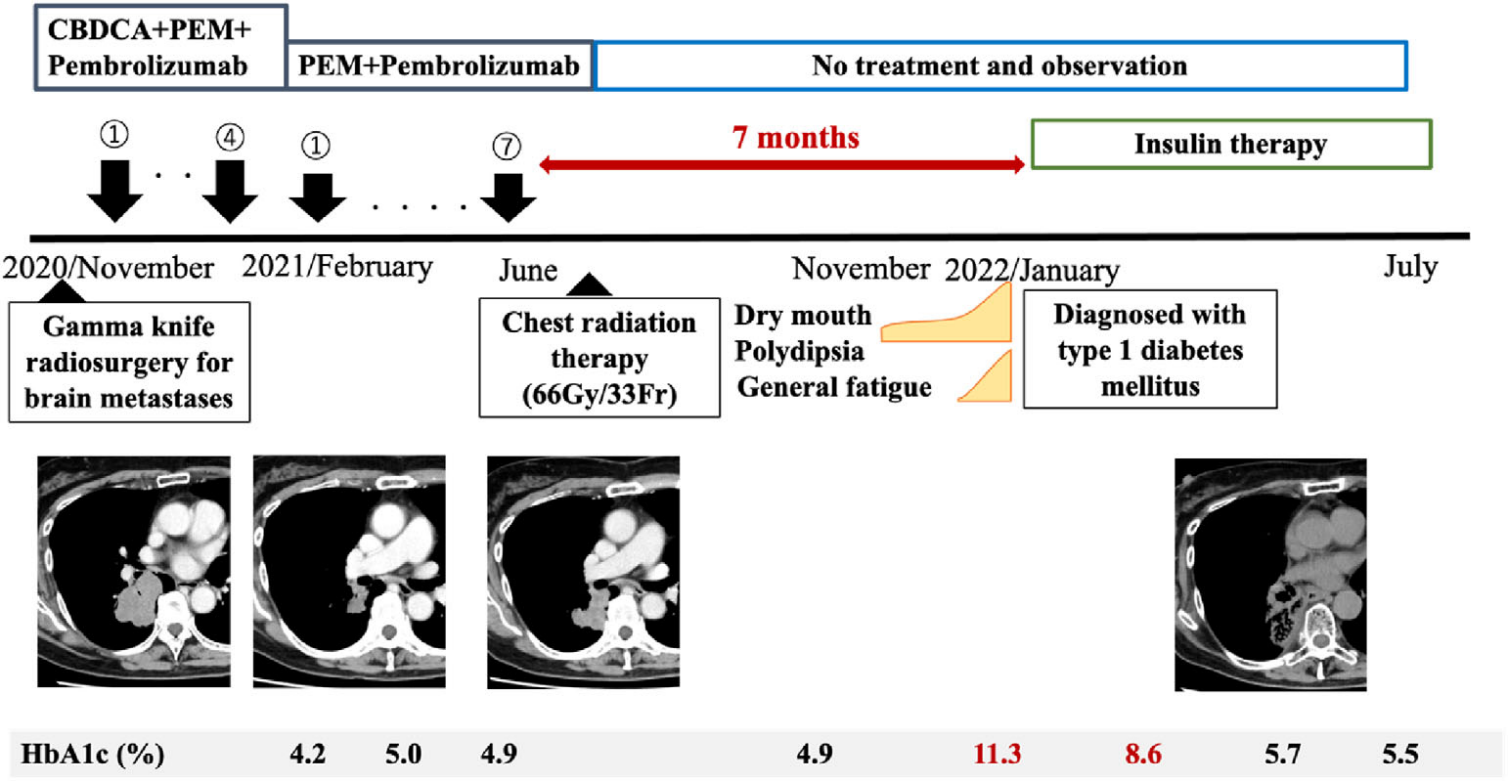
Clinical course of the present case. The patient was diagnosed with type 1 diabetes 7 months after the discontinuation of pembrolizumab. Following the initiation of insulin therapy, the normalization of HbA1c was achieved

A 68‐year‐old woman was referred to our hospital for chest abnormalities. Chest X‐ray revealed a tumor shadow in the right middle lung field. She had no history of smoking or a family history of DM. The Eastern Cooperative Oncology Group performance status was 1. Chest X‐ray (Figure 1a) and contrast‐enhanced computed tomography (CT) showed the tumor in the right lower lobe (Figure 1b) and mediastinal lymph node swelling. Contrast‐enhanced head magnetic resonance imaging revealed a metastatic brain tumor (Figure 1c). Positron emission tomography/CT showed the uptake of fluorodeoxyglucose (FDG) in the tumor and mediastinal lymph nodes, with a maximum standardized uptake value of 19.2 (Figure 1c). Transbronchial tumor biopsy showed adenocarcinoma. No other distant metastases were detected, and the patient was clinically diagnosed with right lower lobe adenocarcinoma cT4N2M1c, stage IVB. A genetic analysis using the Oncomine Dx Target Test Multi‐CDx System revealed that the primary tumor was positive for the human epidermal growth factor receptor 2 gene (ERBB2). Immunostaining for the expression of programmed death ligand 1 (22C3 clones) was negative in tumor cells. Based on these results, combined immunotherapy with carboplatin (AUC 5), pemetrexed (500 mg/m^2^), and pembrolizumab (200 mg) was performed as first‐line therapy. The patient achieved clinical improvements and a partial response. After four courses, maintenance therapy with pemetrexed (500 mg/m^2^) plus pembrolizumab (200 mg/body) was initiated. After the seventh course, maintenance therapy was stopped because only the primary tumor showed local enlargement. Local chest radiotherapy (66 Gy/33 Fr) was performed and revealed a partial response. The patient was followed up without treatment. HbA1c was 4.9% 3 months after the completion of pembrolizumab, and dry mouth and polyuria occurred after 5 months (Figure 2). Seven months later, the patient presented to the emergency room for the exacerbation of thirst, polyuria, and fatigue. Physical examination findings were unremarkable, except for the dry mouth. The patient's weight 6 months previously was 65.7 kg and had decreased to 59.7 kg by the time of her admission (body mass index of 23.6 kg/m^2^). Blood glucose was elevated at 348 mg/dL and HbA1c was 11.3%. Urinalysis revealed significant glycosuria and ketones. An arterial blood gas analysis showed pH 7.257, pCO_2_ 26.5 mmHg, bicarbonate 11.8, base excess −13.6, and an anion gap of 18.2 mmoL/L, which were elevated, suggesting metabolic acidosis. These results were consistent with diabetic ketoacidosis. Urinary C‐peptide was 9.3 μg/day, indicating an endogenous insulin deficiency, and antiglutamic acid decarboxylase antibodies were negative. We did not measure fasting blood insulin or C‐peptide levels or examine other autoantibodies. Neither elevated pancreatic enzymes nor organic abnormalities in the pancreas were noted on echocardiography, therefore we concluded that the patient had developed type 1 DM and diabetic ketoacidosis due to irAE with pembrolizumab. As an initial treatment, the patient received insulin therapy with adequate fluids and electrolyte correction. She is continuing insulin therapy and blood glucose control is excellent. The patient is being monitored for lung cancer and antitumor effects are continuing (Figure 2).

## DISCUSSION

We encountered a lung cancer patient diagnosed with acute‐onset type 1 DM 7 months after the cessation of pembrolizumab. To the best of our knowledge, this is the first case report of acute‐onset type 1 DM 7 months after the discontinuation of pembrolizumab.

ICIs have markedly changed the treatment of lung cancer, and their indications have expanded beyond advanced lung cancer to include adjuvant and neoadjuvant settings.^9^, ^10^, ^11^, ^12^ However, severe irAEs occur in rare cases,^1^ even after the discontinuation of ICIs.^2^, ^3^, ^4^, ^5^, ^6^, ^7^, ^8^ The late‐onset toxicity of ICI therapy has been attracting increasing attention.^13^, ^14^

Type 1 DM is one of the most crucial irAEs caused by ICIs because it progresses rapidly and fatally.^15^, ^16^, ^17^ ICI‐induced DM occurs more frequently with pembrolizumab (2.2%) than with nivolumab (1%).^18^ These values are higher than those expected in spontaneous type 1 DM.^18^ The islet autoantibody positivity rate was reportedly between 40 and 50%, which was not high.^16^, ^17^ ICIs were shown to still bind to lymphocytes 20 weeks after the administration of the last dose.^19^ These findings provide insights into the occurrence of irAEs after the discontinuation of ICIs.

## CONCLUSION

We herein presented a lung cancer patient diagnosed with acute‐onset type 1 DM 7 months after the cessation of pembrolizumab. Clinicians need to consider the possibility of irAEs in patients receiving ICIs, even after their discontinuation.

## CONFLICT OF INTEREST

The authors declare no conflicts of interest.
